# Computational pathology in the age of artificial intelligence – embrace not fear

**DOI:** 10.1002/2056-4538.70049

**Published:** 2025-09-22

**Authors:** Alfonso Tan‐Garcia, Tzy Harn Chua, Wei‐Qiang Leow

**Affiliations:** ^1^ Department of Anatomical Pathology Singapore General Hospital Singapore; ^2^ Duke‐NUS Medical School Singapore; ^3^ School of Biological Sciences Nanyang Technological University Singapore

**Keywords:** computational pathology, histopathology, artificial intelligence, machine learning, diagnostic, research

## Abstract

Anatomical pathology has traditionally relied on the interpretation of histomorphological features under a light microscope by trained pathologists for diagnosis. Technological advancements have enabled the digitisation of tissue slides to produce high‐resolution whole slide images, heralding the era of digital pathology (DP). Many laboratories around the world have incorporated DP into their routine workflows owing to the myriad applications it offers in facilitating tumour board discussions, remote reporting, teaching, and research. Most significantly, DP has engendered the field of computational pathology, a novel branch of histopathology incorporating artificial intelligence (AI) models. Computational pathology has been utilised in histomorphological quantification and diagnostic, predictive, and prognostic applications due to its potential to improve diagnostic accuracy, personalise treatment, and streamline workflows. Here, we highlight the work of Meier *et al*, Shen *et al*, and Lee *et al*, published in this journal in recent years, as they apply AI models to predict survival and treatment responses in gastric cancer, breast cancer, and diffuse large B‐cell lymphoma, respectively. Collectively, these studies illustrate various approaches to incorporating AI into the DP pipeline and their potential clinical applications. Issues related to diagnostic accuracy, cost, patient confidentiality, and regulatory ethics still need to be addressed within the field. Despite this, the overall sentiment among pathologists is one of cautious optimism.

## Introduction

Anatomical pathology is a branch of medicine that deals with the interpretation of histomorphological features to render a diagnosis that aids in the clinical management of a patient. Historically, since the 19th century, light microscopy has been the gold standard for histological examination of tissue sections on a glass slide. In the 1960s, with technological advancements, Prewitt and Mendelsohn [1] were able to scan images of a microscopic field, thus paving the way for the advent of digital pathology (DP). It was not until 1986 that Ronald Weinstein coined the term ‘telepathology’, referring to the transmission of digital images over a long‐range network [2]. Since its introduction in 1990, whole slide scanners have allowed the digitalisation of entire glass slides with several layers of magnification into whole slide images (WSIs), transforming the conventional microscope into a virtual microscope [3, 4]. In 2017, the IntelliSite Pathology Solution (PIPS, Philips Medical Systems, Best, Netherlands) received US Food and Drug Administration (FDA) approval as a comprehensive digital pathology WSI system [5]. While some institutions have converted fully to DP for routine reporting [6], many more around the world have made significant changes to incorporate DP into their routine workflows [3, 7], carefully navigating issues such as the cost of slide scanners, data storage, and data security, among others [4, 8, 9]. Importantly, a multicentre study found that the primary diagnostic performance of DP WSI was not inferior to that of conventional microscopy [10]. The implementation of DP has facilitated remote reporting, external consultations, teaching, and research [4].

In recent years, one of the most important technological advances has been the application of artificial intelligence (AI) in DP, creating the novel field of computational pathology. Computational pathology involves the use of AI algorithms that can extract histomorphological information from a digital image and, at the most basic level, aid in routine diagnostic work, which can at times be fraught with interobserver discrepancies [3]. For example, it can be used to grade tumours and identify mitotic figures and acid‐fast bacilli [11]. AI can also reduce the workload of pathologists and improve accuracy and consistency of diagnoses, minimising errors and improving patient care [7, 12]. A systematic review and meta‐analysis of the diagnostic accuracy of AI models applied to WSI for detecting disease across all disease types reported a high sensitivity and specificity of 96.3% and 93.3% respectively [13], providing strong evidence that AI algorithms can assist in clinical diagnosis. Many research initiatives have focused on the detection and grading of prostate cancer due to its high prevalence and interobserver variability, culminating in US FDA approval in 2021 for the clinical use of Paige Prostate Detect, an assistive AI diagnostic tool [14, 15]. Recently in April 2025, Paige PanCancer Detect, which assists in the detection of foci suspicious for cancer in multiple tissues and organs, was granted approval [16, 17]. On a wholistic level, AI algorithms can be used to discover novel biologically and clinically relevant information by analysing histological features of a tumour and/or its microenvironment in association with vast amounts of genomic, transcriptomic, proteomic, and clinical data, which would otherwise not be possible by manual interpretation [7, 8]. In August 2025, ArteraAI Prostate, a multimodal AI‐powered tool that combines clinical data and histological images, received *de novo* authorisation by the US FDA for use in prognosticating the 10‐year risk of distant metastasis and prostate‐cancer‐specific mortality in patients with non‐metastatic prostate cancer [18]. AI algorithms have also been developed to interrogate the non‐neoplastic stromal compartments, revealing new insights into disease biology and prognosis in breast, oropharyngeal, prostate, and non‐small‐cell lung cancers [3, 19].

## Technical workflow

**Figure 1:**
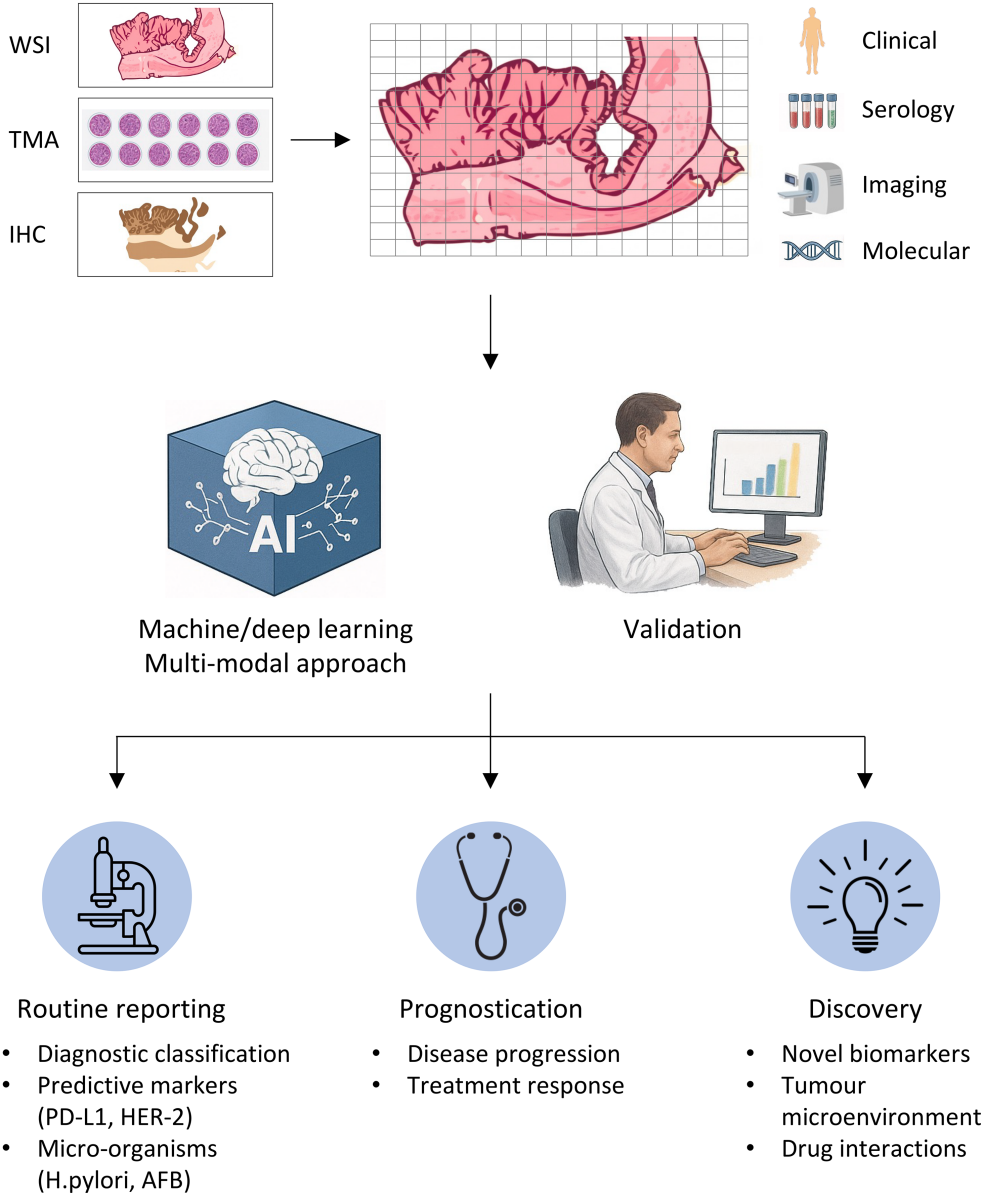
Schematic workflow illustrating incorporation of AI into histopathology and its applications. (Top panel) Digitally scanned H&E‐stained tissue images in the form of whole slide images (WSIs) or tissue microarrays are selected for analysis. Depending on the application or research question, immunostained slides can also be used. Digital slide images are divided into smaller tiles for incorporation into the AI model during the training phase. Additional data can also be fed into the AI pipeline including clinical, imaging, and serological findings and molecular data at the genomic, epigenomic, transcriptomic, or proteomic level. (Middle panel) Various machine and/or deep learning AI models can be adopted in a singular or multi‐modal approach, the most common being convolutional neural networks. The trained AI models need to undergo rigorous clinical validation before incorporation into routine workflows. (Lower panel) The applications of AI in routine reporting, disease prognostication, and research. AFB, acid‐fast bacilli; IHC, immunohistochemistry; TMA, tissue microarray. Created with the aid of OpenAI's ChatGPT.

The proposed pipeline for implementing computational pathology is outlined in a schematic diagram (Figure 1). The workflow begins with the selection of tissue types containing regions of interest, ranging from large tissue sections from excisions to small tissues obtained from core needle biopsies. The use of tissue microarrays (TMA), which involves the creation of a composite paraffin block composed of multiple small tissue cores from different cases, allows for high‐throughput analysis and cost effectiveness [20]. Next, the type of tissue stain will have to be considered. Traditional haematoxylin‐eosin (H&E) staining forms the backbone of histopathology, providing details on tissue architecture and cytomorphology. The incorporation of immunohistochemical (IHC) stains into the analysis provides additional information related to tumour phenotype and tumour microenvironment, depending on the markers chosen. The slides are then digitally scanned to create WSIs. Most modern digital scanners have the ability to scan up to ×40 magnification and at multiple z‐stack levels, producing high‐resolution images (commonly 100k × 100k pixels) with colour information [7]. The image tiles are subsequently used to train the AI algorithm. In addition, clinical, imaging, and serological findings as well as molecular data at the genomic, epigenomic, transcriptomic, or proteomic level can also be used to train the model to potentially uncover novel associations between individual tiles and other clinical or molecular parameters.

Image analysis can be performed by either machine learning (ML) or deep learning (DL) AI algorithms. ML algorithms such as support vector machines (SVM) and random forest (RF) learn associations between predefined image features based on training data fed into them [8]. Conversely, DL algorithms such as convolutional neural networks (CNN) automate both feature extraction and learning of associations and are ideal for computer vision and image classification [8]. CNNs comprise multiple layers of artificial neural networks mimicking the human neural architecture and work by hierarchically deconstructing images into low‐level spatial cues which are then aggregated into high‐order structural relationships to identify features of interest [3]. Other DL models such as generative adversarial network, fully convolutional network and recurrent neural network are also available [3]. WSIs cannot be directly incorporated into CNNs due to their large size and are divided into patches or tiles, allowing for more detailed image analysis [21]. Patch‐based aggregation methods then allow the algorithm to make predictions based on entire slides [21]. However, not every tile on a slide will reflect the ground truth label; instead, only the plurality of tiles generated from a WSI represents a specific label [8]. Therefore, multiple instance learning (MIL), coupled with an ‘attention’ approach in which the algorithm applies a weighted average to each tile, gives a more accurate interpretation [8].

Another consideration when selecting AI algorithms is the level of supervision required. In a supervised approach, slides are fully annotated by pathologists before being used for training the algorithm. This approach is labour‐intensive, time‐consuming, and unscalable [21]. In a weakly supervised approach, the algorithm can learn from incomplete or imprecise labels such as slide‐level rather than tile‐level labels [9]. For example, MIL can learn to associate individual tiles that contain tumour even though only the slide is labelled as tumour, without specifying tumour boundaries. In an unsupervised approach, the algorithm discovers patterns from unlabelled slides on its own and is useful for exploratory analysis of large datasets [8, 21]. Self‐supervised algorithms such as self‐distillation with no labels (DINO) are pre‐trained using unlabelled slides to learn patterns and subsequently fine‐tuned with a supervised dataset in a two‐step methodology that has been proven to be superior to a single‐step supervised approach [8]. After training, the performance of the AI algorithms should be clinically evaluated on validation cohorts.

Several publications in *The Journal of Pathology: Clinical Research* over the years aptly demonstrate the pipeline for implementing AI in pathology research. Meier *et al* evaluated TMAs of both H&E and IHC stains for CD8, CD20, CD68, and Ki‐67 in a cohort of 248 gastric cancer patients using a weakly supervised CNN‐based algorithm [22]. They found that CNN‐derived risk scores provided additional prognostic value compared to the gold standard TNM staging and that IHC‐derived risk scores provided better prognostication than H&E‐derived risk scores. In addition, the presence of B‐cell clusters and Ki‐67‐positive subregions was associated with low risk of cancer‐specific deaths. The incorporation of IHC slides for T‐cell, B‐cell, and macrophage markers provided novel biological insights into the role of the tumour immune microenvironment and its association with disease prognosis, potentially uncovering new treatment strategies. In contrast, Shen *et al* analysed H&E slides of core biopsies of 310 invasive breast cancer tumours and successfully predicted the efficacy of neoadjuvant chemotherapy with an accuracy of 95% [23]. Interesting, they used a pipeline of three independent AI models, each focusing on different tumour features. This included a CNN model to evaluate structural atypia and SVM and RF models to evaluate nuclear atypia. Lee *et al* retrospectively analysed H&E slides from 216 patients with diffuse large B‐cell lymphoma treated with immunochemotherapy using the self‐supervised DINO model [24]. The histological features extracted by the algorithm showed significant correlations with clinical parameters and predicted relapse‐free survival, which was corroborated by external validation with additional datasets from The Cancer Genome Atlas (TCGA). In summary, these publications leveraged computational pathology to predict survival and treatment responses by incorporating multiple parameters, including clinical data and histomorphological and IHC features. Therefore, AI heralds a new age of wholistic patient care whereby multiple sources of data generated in the course of clinical care can be concatenated and interrogated simultaneously to devise the most appropriate management plan.

## Limitations and future work

The development and implementation of computational pathology has several limitations. The laboratory information system has to be integrated with DP and laboratory workflows optimised to reduce disruption to routine reporting. Laboratories have to be equipped with digital scanners capable of capturing high resolution WSI and adequate data storage devices for the large files generated [7, 9]. There must be comprehensive regulations in place to protect confidential patient data in the form of encryption or access control [21]. The means by which AI algorithms arrive at their conclusions is not well understood, even to their developers, thereby earning the label of ‘black boxes’ [7]. In the medical field, trust and transparency in the AI algorithm are indispensable to justify medical decision‐making and comply with regulatory requirements. Explainable AI, or XAI, is a branch of machine learning which seeks to provide human‐interpretable explanations for how complex AI algorithms generate specific predictions [25]. In pathology, XAI methods such as prototypes, saliency maps, counterfactuals, concept attribution, and trust scores can be incorporated into AI algorithms. In addition to explainability, AI algorithms should also provide for causability, or the quality of explanations, in the form of a human‐AI interface which allows the user to request explanations on demand and probe the reasons underlying the algorithm's prediction [25]. While research into XAI methods is ongoing, legal and regulatory guidelines are necessary to protect patients. For example, the EU's new General Data Protection Regulation stipulates that individuals shall have the right not to be subject to decisions based solely on automated processing [7]. Several prominent organisations including the World Health Organisation [26], Royal College of Pathologists (UK) [12] and the Royal College of Pathologists of Australasia [27] have released guidelines on the incorporation of AI in pathology emphasising the need for safe and ethical implementation. Finally, the notion that AI will replace pathologists is unfounded; a more likely scenario is one where the pathologist who uses AI will replace the pathologist who does not.

## Author contributions statement

All authors drafted and reviewed the manuscript. The figure was prepared by AT‐G with review from all authors.
